# Tankyrase Inhibitors Stimulate the Ability of Tankyrases to Bind Axin and Drive Assembly of β-Catenin Degradation-Competent Axin Puncta

**DOI:** 10.1371/journal.pone.0150484

**Published:** 2016-03-01

**Authors:** Estefania Martino-Echarri, Mariana G. Brocardo, Kate M. Mills, Beric R. Henderson

**Affiliations:** Centre for Cancer Research, The Westmead Institute for Medical Research, The University of Sydney, Westmead, NSW 2145, Sydney, Australia; University of Washington, UNITED STATES

## Abstract

Activation of the wnt signaling pathway is a major cause of colon cancer development. Tankyrase inhibitors (TNKSi) have recently been developed to block the wnt pathway by increasing axin levels to promote degradation of the wnt-regulator β-catenin. TNKSi bind to the PARP (poly(ADP)ribose polymerase) catalytic region of tankyrases (TNKS), preventing the PARylation of TNKS and axin that normally control axin levels through ubiquitination and degradation. TNKSi treatment of APC-mutant SW480 colorectal cancer cells can induce axin puncta which act as sites for assembly of β-catenin degradation complexes, however this process is poorly understood. Using this model system, we found that siRNA knockdown of TNKSs 1 and 2 actually blocked the ability of TNKSi drugs to induce axin puncta, revealing that puncta formation requires both the expression and the inactivation of TNKS. Immunoprecipitation assays showed that treatment of cells with TNKSi caused a strong increase in the formation of axin-TNKS complexes, correlating with an increase in insoluble or aggregated forms of TNKS/axin. The efficacy of TNKSi was antagonized by proteasome inhibitors, which stabilized the PARylated form of TNKS1 and reduced TNKSi-mediated assembly of axin-TNKS complexes and puncta. We hypothesise that TNKSi act to stimulate TNKS oligomerization and assembly of the TNKS-axin scaffold that form puncta. These new insights may help in optimising the future application of TNKSi in anticancer drug design.

## Introduction

β-catenin is the central activator of the wnt signaling pathway and transduces signal from the plasma membrane to the nucleus. This pathway is important for development and its aberrant activation is a major cause of colorectal cancer (CRC) [1, 2]. β-catenin levels need to be tightly regulated under normal cell conditions and this is achieved by the β-catenin degradation complex [3]. This complex is formed by several core proteins including the protein scaffolds axin and adenomatous polyposis coli (APC), and two kinases CK1 and GSK3-β. β-catenin is sequestered to this multiprotein site where it is phosphorylated at the N-terminal region (Ser33/Ser37/Thr41) by CK1 and GSK3-β, marking it for degradation by the proteasome [4]. The disruption through gene mutation of these key β-catenin degradation components is known to increase the protein levels of β-catenin which then translocates to the nucleus and activates LEF-1/TCF transcription factors and gene networks that promote CRC development. There is strong interest in developing anti-cancer approaches that target the wnt pathway and the corresponding increase in β-catenin protein for clinical use.

Tankyrases (TNKSs) 1 and 2 are poly(ADP)ribose polymerases (PARPs) which have gained attention in recent years as promising drug targets due to their role in down-regulating axin and stabilizing β-catenin. Indeed, TNKS inhibitors (TNKSi) have been developed that can disrupt the wnt pathway by causing an increase in axin and subsequent decrease in β-catenin levels [5]. Certain TNKSi have been shown to promote the formation of large β-catenin degradation complexes, visible under the microscope as axin-positive puncta in APC-mutant SW480 CRC cells [6]. Axin is well known for its capacity to self-oligomerize through its DIX domain and to act as a scaffold to promote hetero-oligomerization with other β-catenin degradation complex components [7, 8]. Axin puncta are thought to be sites for large macromolecular assemblies that comprise protein factors such as APC and GSK-3β required for sequestering and targeting β-catenin for degradation [9].

The addition of poly(ADP)ribose (PAR) groups to axin by TNKS label it for degradation by the proteasome [10–12]. TNKS are also known to regulate their stability and activity through auto-PARylation [5, 13]. Specific TNKSi have been designed to block the activity of TNKSs by binding to the catalytic domain in the NAD pocket. The pocket is formed by two subunits and inhibitors for both have been developed. XAV939 is directed against the nicotinamide subsite which is conserved amongst different PARP proteins, whilst G007-LK and IWR-1 are directed against the adenosine subsite and are more potent and specific inhibitors of TNKSs [14]. There are opposing results in the literature for the action of these TNKSi. Some studies reported promising results in APC mutated CRC cell lines [15, 16], whilst others showed that nuclear β-catenin bound to LEF-1/TCF transcription factors would be protected from degradation such that TNKSi treatment would not be effective [6]. These conflicting reports make it clear that a better understanding of how TNKSi function to down-regulate wnt/β-catenin signaling at a molecular level is required, in order to develop future combinatorial therapies based on these small molecule inhibitors.

## Material and Methods

### Cell culture, drug treatments and transfection

The primary cell line used here are human SW480 colorectal cancer cells (APC protein truncated at amino acid 1337), purchased from the commercial cell repository CellBank Australia (Sydney) in August 2014. The cells were confirmed to express key markers unique to this cell line (ie. nuclear beta-catenin and mutant truncated APC) and were verified mycoplasma negative. Some control experiments were performed using human embryonic kidney HEK293T cells and mouse NIH 3T3 fibroblasts (obtained from the Westmead Institute cell database, confirmed mycoplasma negative and described previously [17]). Cells were cultured in DMEM supplemented with 10% FBS, 40 mM L-glutamine, 100 units/ml Penicillin and 100 μg/ml Streptomycinhttps://www.thermofisher.com/us/en/home/life-science/cell-culture/mammalian-cell-culture/antibiotics/penicillin-streptomycin-mixtures.html at 37°C in a 5% CO2-humidified incubator. Cell lines were treated with different proteasome inhibitors: 20 μM MG132 (Merck-Millipore, Germany) for 6 h, if not stated differently, and 10 μM Bortezomib (Selleckchem, USA) for 4 h. Cells were treated with 2.5 μM XAV939 and 5 μM IWR-1 (Sigma-Aldrich, USA) and 5 μM G007-LK (Merck-Millipore, Germany) to inhibit TNKS activity. Transfections were carried out using Lipofectamine 2000 (Invitrogen, USA) according to manufacturer instructions. We transfected cells with the plasmid pβ-catenin-WT-GFP [17]. A combination of 4 specific siRNAs were used to silence human AXIN1 (SMARTpool ON-TARGETplus; Dharmacon, USA). We purchased siRNA sequences for human TNKS that were previously described [5]. For each TNKS knockdown we used a combination of two siRNA and thus a total of four siRNA sequences were employed to silence both TNKS-1 and -2 simultaneously.

### Western blotting

Cells were lysed using RIPA buffer containing protease inhibitor cocktail Complete (Sigma-Aldrich, USA) and 0.1 M of sodium orthovanadate (Sigma-Aldrich, USA). Samples were boiled for 5 min at 95°C and loaded on to 7.5% or 10% SDS-acrylamide gels. Gels were blotted on nitrocellulose membranes (Merck-Millipore, USA) and blocked for 1 h at room temperature with 3% skim milk in 0.2% TBST. Primary antibodies were incubated overnight at 4°C. Anti-mouse/rabbit/goat horseradish peroxidase conjugated (HRP) secondary antibodies (Sigma-Aldrich, USA) were incubated for 1 h at room temperature. Western Bright ECL Western Blotting Kit (Advansta, USA) was used to detect chemiluminescence, and blots were imaged using the ChemiDoc MP Imaging system (Bio-Rad, USA). Band intensities were measured using Image Lab software from Bio-Rad.

### Detergent extraction assay

The following buffers were pre-warmed at 37°C before start; MT-stabilizing buffer (130 mM HEPES, pH 6.9, 2 mM MgCl_2_, 10 mM EGTA), and MT-fixation buffer (130 mM HEPES, pH 6.9, 2 mM MgCl_2_, 10 mM EGTA, 120 mM sucrose and 3.7% formalin). Cells were washed twice with MT-stabilizing buffer, then incubated with MT-stabilizing buffer plus 0.2% Triton X-100 for 5 min at 37°C to extract soluble proteins. Cells were then washed twice with MT-stabilizing buffer and fixed with MT-fixation buffer for 30 min at 37°C. Cells were then stained for microscopy as outlined below.

### Immunofluorescence and cell imaging

Cells for immunofluorescence were fixed with PBS-3.7% formalin for 20 min and cells were subsequently permeabilized with PBS-0.2% Triton X-100 solution for 10 min. Non-specific interactions were blocked with PBS-3% BSA. Primary and secondary antibody incubations were performed in PBS-3% BSA for 1 h at room temperature. Fluorescence detection was performed using Alexa-Fluor 488/594/647 (Invitrogen, USA) secondary antibodies and Hoechst dye (H6024, Sigma Aldrich, USA) nuclear stain. The following primary antibodies were used for western blotting and immunofluorescence: Axin (AF3287, R&D Systems, USA), axin (C76H11, 2087, CST, USA), β-catenin (610153, Becton Dickinson, USA), β-actin (A5316, Sigma-Aldrich, USA), TNKS (H-350) (sc-8337, SCBT, USA), GSK3β (L-17) (sc-8257, SCBT, USA), APC (H-290) (sc-7930, SCBT, USA), phosho β-catenin (9561, CST, USA), non-phosho β-catenin antibodies (8814, CST, USA) and α-tubulin (T6074, Sigma-Aldrich, USA).

Immunofluorescence microscopy cell imaging acquisition was performed with an Olympus DeltaVision core deconvolution microscope with SoftworxResolve 3D software. Images were taken for every field using a 40x objective and deconvolution was used to process the images.

### Immunoprecipitation (IP) and cell fractionation

Cells were lysed using the IP lysis buffer (87787, Thermo Fisher Scientific, USA). Protein extracts were quantified and 1 mg of protein was incubated overnight under constant rotation with 2 μg of the required antibody (PAR antibody, 4335-MC-100-AC, Trevigene, USA; Axin, AF3287, R&D Systems, USA). 30 μl of protein-G agarose (Roche, Switzerland) beads were added to the samples and incubated for a further 3 h at 4°C under constant rotation. Beads were washed three times with 400 μl of the IP lysis buffer to remove non-specific binding. Then the beads were incubated with 30 μl of SDS sample buffer and boiled for 5 min at 95°C. Proteins were separated by SDS-PAGE and blotted onto nitrocellulose membranes.

To obtain cell fractions enriched for soluble and insoluble protein, two methods were employed. Cells (untreated or treated) were trypsinized and lysed normally in RIPA buffer to obtain a soluble lysate, or an enriched insoluble protein pool was obtained by lysing and extracting cells on the plate with SDS-PAGE (Laemmli) sample buffer for 15 min and collecting the extracted cell material using a cell scraper. All lysates were analyzed by SDS-PAGE. Alternatively (see Supplementary data), 1 x 10^6^ cells were treated with 200 ml of ice-cold 0.1% Triton X-100 in CSK buffer for 10 min at 4^o^ C. The cells were pelleted by low-speed centrifugation (5 min at X1000 g) and the supernatant represents the soluble fraction. The pellet with insoluble protein was solubilized in 90 ml of RIPA buffer for 20 min and the protein suspension centrifuged at maximum speed to obtain the insoluble fraction pellet analyzed by SDS-PAGE.

### Statistics

Data analysis was performed in GraphPad Prism 5 and where appropriate included use of Student’s t-test and one-way or two-way ANOVA. Differences were considered to be statistically significant if P < 0.05 (*), P < 0.01 (**) and P < 0.001 (***).

## Results and Discussion

### TNKS inhibitors induce formation of endogenous axin puncta that correspond to β-catenin degradation sites

**Fig 1:**
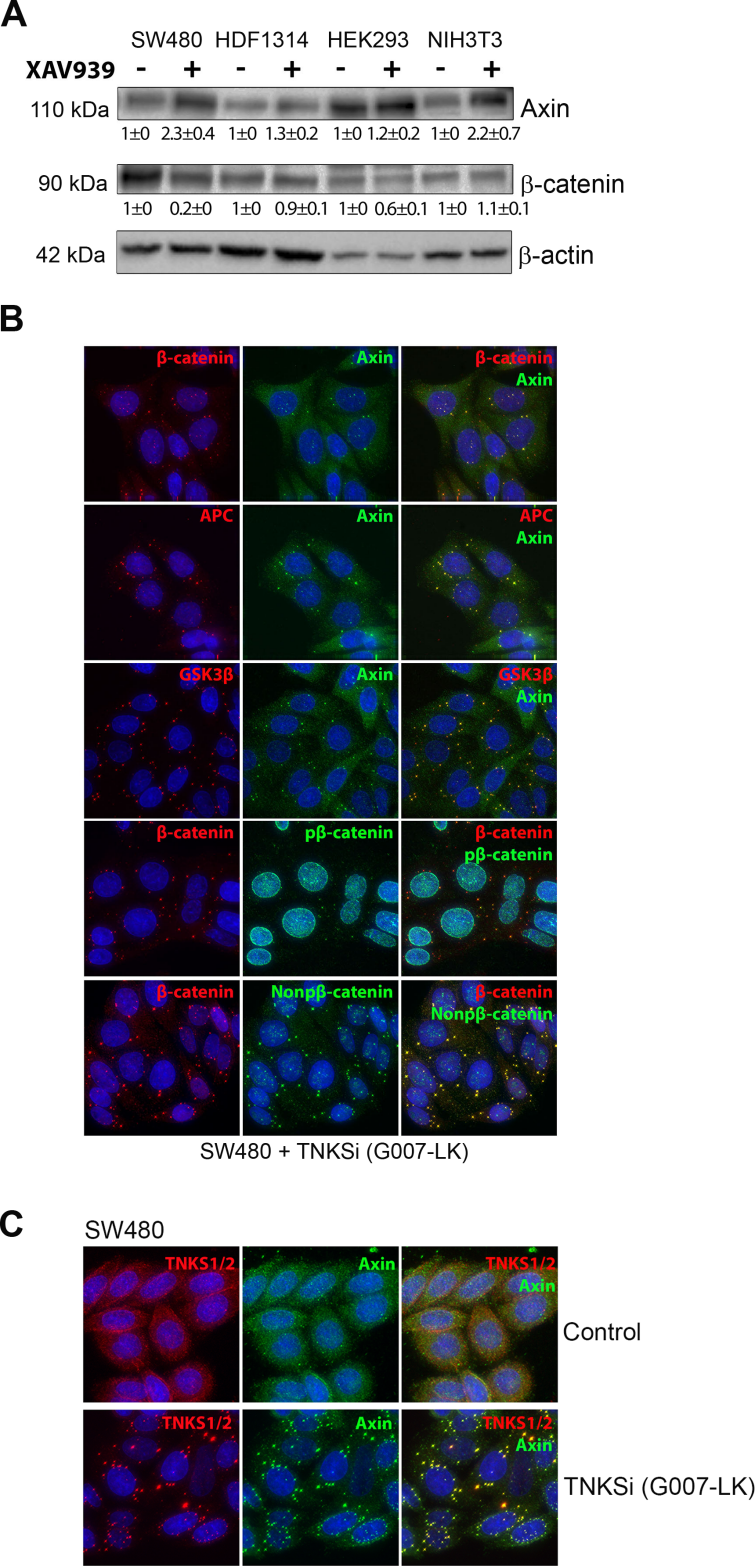
TNKSi treatment can induce axin expression and axin puncta associated with β-catenin degradation. **A.** Western blot analysis of different cell lines treated +/- for 24 h with 2.5 μM of the TNKSi XAV939 revealed an increase in axin expression in different cell lines, and rescue of the degradation of β-catenin in SW480 CRC cells where the wnt pathway is disrupted by APC mutation. β-actin levels are shown as loading controls. Quantification of band intensities from two separate blots was performed and normalized to actin. The values shown are mean ± SD. **B.** SW480 cells were treated with 5 μM of the TNKSi G007-LK for 24 h, then immunostained with specific antibodies to detect different components of the β-catenin degradation complex located at axin puncta. The following combinations of proteins were co-stained: axin and total β-catenin, APC and Axin, GSK3β and Axin and phosphorylated and non-phosphorylated β-catenin. Cells were imaged using a DeltaVision microscope system. **C.** SW480 control cells or cells treated with 5 μM of the TNKSi G007-LK for 24 h were stained for fluorescence. In control cells a disperse staining pattern is observed whereas in TNKSi treated cells the TNKS-1 and -2 (red) specifically colocalize at TNKSi induced axin puncta (green) as shown by immunofluorescence microscopy.

TNKSi have previously been shown to disrupt wnt signaling by inducing β-catenin turnover, even in APC-mutant SW480 CRC cells where β-catenin is over-expressed [5, 9]. Here we used western blot analysis to test the effectiveness of TNKSi (XAV939) on axin protein accumulation in different cell lines (Fig 1A). The drug caused some increase of axin in most cell lines, and in SW480 cells which express high levels of β-catenin and have poor axin expression, the drug had a very pronounced effect stimulating axin levels and down-regulating β-catenin (Fig 1A). The ability of TNKSi to restore degradation of β-catenin in CRC cells has been observed elsewhere [16]. This observation correlates with the induction of β-catenin degradation sites, otherwise known as axin-positive puncta, in SW480 cells by TNKSi (Fig 1B) (note, for simplicity we will refer to the axin puncta as β-catenin degradation sites, although a pool of the β-catenin may be modified and released from puncta for degradation elsewhere in the cell). We detected low levels of both N-terminal phosphorylated and non-phosphorylated β-catenin at axin puncta in SW480 cells, consistent with the role of these puncta in active β-catenin turnover ability. The puncta were induced by different TNKSi directed against either the nicotinamide (XAV939) or the adenosine (IWR-1 and G007-LK) subsites (Fig 1 and S1 Fig). In addition, we corroborated recent observations [9] that TNKSs are associated with the TNKSi-induced axin puncta (Fig 1C). In NIH 3T3 cells that efficiently degrade β-catenin, we could detect axin puncta forming after TNKSi treatment and such puncta displayed little detectable endogenous β-catenin, presumably because it is actively degraded; only when β-catenin was overexpressed as a GFP-fusion was it then visualized at the axin puncta of those cells (S2 Fig).

### Expression of tankyrases 1 and 2 is required for TNKSi-induced formation of axin puncta

**Fig 2:**
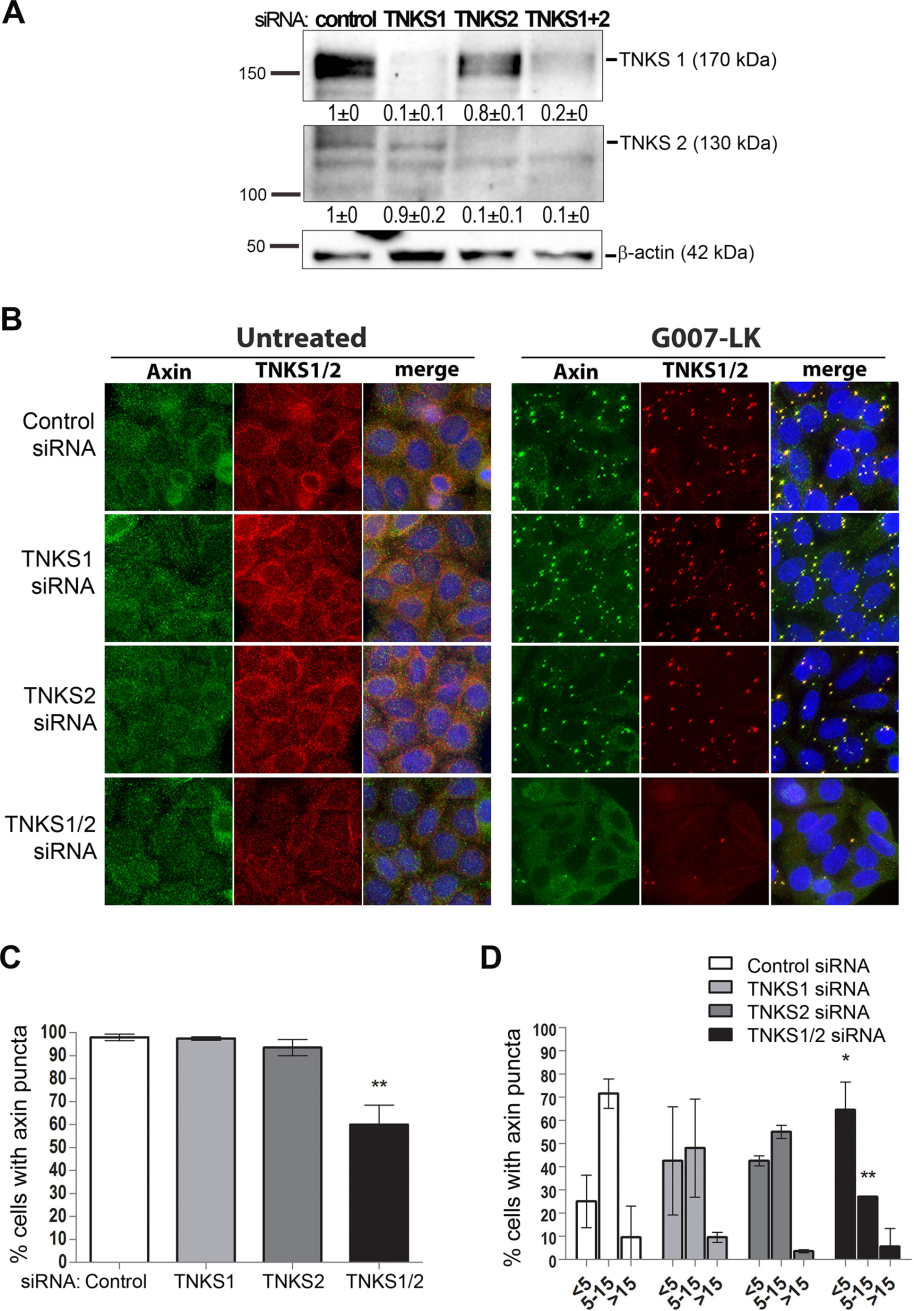
TNKS expression is required for TNKSi-dependent assembly of axin puncta. The effect of silencing expression of TNKS1, TNKS2 or both (TNKS1/2) by siRNA was examined on TNKSi induced puncta formation in SW480 cells. **A.** Western blot and quantification showing the differences in TNKS expression after knockdown of the individual TNKSs. The band intensities represent the mean ± SD (normalized to actin) of two independent experiments. **B.** Immunofluorescence staining of SW480 cells untreated or treated for 24h with 5 μM of the TNKSi G007-LK. The individual knockdown of TNKS1 or TNKS2 did not significantly alter axin puncta formation, but the combined silencing of both TNKS1 and TNKS2 (siTNKS1/2) caused a clear reduction of cells showing axin and TNKS-positive puncta after G007-LK treatment, as shown by the cell images. **C.** A general scoring for axin puncta formation by microscopy revealed a ~40% decrease in the % cells showing visible axin puncta but only after silencing of both TNKS-1 and -2 (one-way anova bonferroni post-test, ** p<0.01 shows significance in comparison to the control). Only small differences were observed after knockdown of the individual TNKS. **D.** A more detailed comparison of the impact of TNKS expression on puncta was performed by scoring the range of puncta present per cell (categories: <5, 5–15 or >15 puncta). Here small reductions in the number of puncta per cell were seen with the individual TNKS siRNAs, but again only the double knockdown caused a significant reduction (two-way anova bonferroni post-test * p<0.05 and ** p<0.01 shows significance relative to control) in the number of puncta per cell.

Since the TNKS inhibitors, which block the poly(ADP)ribose catalytic domain, can induce formation of axin puncta we asked whether the loss of expression of either TNKS1, TNKS2 or both, also drives axin puncta assembly in SW480 cells. We knocked down TNKS1 and TNKS2 expression by siRNA treatment (see western blot in Fig 2A). Surprisingly, we found that knockdown of TNKS1 and/or TNKS2 did not phenocopy the effect of TNKSi on formation of axin puncta. As shown in Fig 2B (left panel) the silencing of TNKS1, 2 or 1+2 was not sufficient to induce endogenous axin puncta, compared with the positive control of TNKSi treatment (Fig 2B TNKSi treatment, right panel). The TNKS antibody used detects both TNKS-1 and -2. Thus, loss of TNKS1/2 expression does not cause formation of axin puncta.

Since the TNKSs are thought to associate with axin at puncta [9], we next tested the possibility that they actually contribute to axin puncta formation, albeit when they are in an inactivated (ie. unable to PARylate) form. We therefore silenced TNKS1 and/or TNKS2 and tested the effect on TNKSi induction of axin puncta (see Fig 2B, right panel). We found that silencing of both TNKS1 and TNKS2 strongly reduced the induction of axin puncta, whereas the individual knockdown of either tankyrase alone had only a minimal effect (quantification shown in graphs in Fig 2C and 2D). This result supports previous evidence showing that TNKSs display some redundancy during mouse embryonic development [18]. Moreover, our findings further suggest that both TNKS1 and TNKS2 can prime axin puncta formation but only when their PAR activity is blocked by inhibitor treatment. This novel finding is consistent with recent data that identified some association of axin and TNKS oligomeric filaments by electron microscopy [9].

### Inactivation of tankyrases 1 and 2 stimulates their binding, and that of β-catenin, to axin

**Fig 3:**
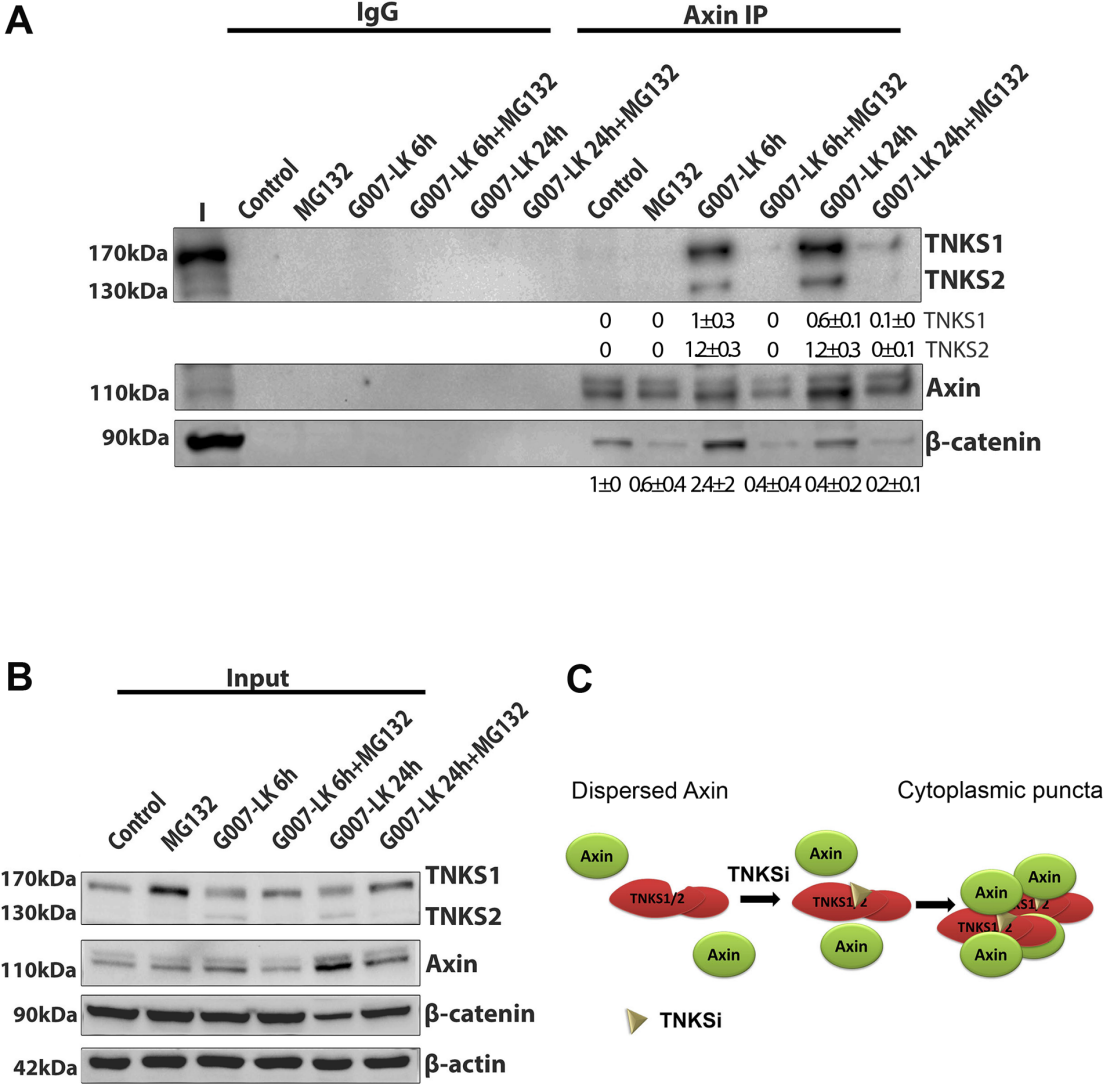
TNKSi induces the binding of TNKSs to axin. **A.** SW480 cells were untreated or treated for 6 h or 24 h with 5 μM G007-LK (+/- 6 h with 20 μM MG132), then cell extracts were harvested and subjected to immunoprecipitation (IP) analysis with an axin antibody to detect axin-protein complexes. The immunoprecipitates were separated by SDS-PAGE and analysed by western blot. As shown, an increase in binding of both TNKS-1 and -2 to axin was observed after TNKSi treatments. In addition, an increase in β-catenin binding to axin was seen after 6 h treatment. In the presence of MG132, the induction of TNKS-axin complexes by G007-LK was blocked as shown. Quantification of axin-bound TNKS-1, TNKS-2 and β-catenin band intensities from two separate experiments are shown (mean ± SD). Values are corrected towards the total axin pulled down per sample. **B.** Western blot of total protein extract demonstrates that total levels of TNKSs were not modified by TNKSi treatment, but displayed a modest increase after 6 h MG132. The G007-LK treatment did modestly increase axin levels (and decrease β-catenin levels after 24 h treatment) as expected. This control blot indicates that the strong induction of TNKS-axin complexes above is not due to changes in protein expression. **C.** A diagram outlining how these novel effects of TNKSi on axin-TNKS complexes may contribute to axin/TNKS positive puncta formation in the cell.

Axin puncta are composed of multiple copies of large macromolecular axin oligomer complexes [7, 8]. Therefore we tested the possibility that tankyrase, which also has been reported to form oligomers [19, 20], becomes associated with axin complexes after TNKSi treatment. We treated SW480 cells with TNKSi (G007-LK) and performed immunoprecipitation of endogenous axin complexes using a specific antibody. As shown in Fig 3A, axin was pulled down effectively but there was very little TNKS bound to axin in untreated (control) cells. Following 6 h TNKSi treatment, however, a significant pool of both TNKS1 and TNKS2 became bound to axin, and this was further increased after 24 h treatment. Consistent with the role of these complexes in β-catenin turnover, β-catenin association with axin complexes also increased after TNKSi treatment (Fig 3A). Under these conditions, western blot revealed that TNKSi treatment did not significantly alter TNKS protein levels (although in this experiment MG132 stabilized TNKS1), while axin protein levels did progressively increase along with a progressive decline in β-catenin levels (Fig 3B). This experiment was also performed in HEK293T cells (S3A Fig) yielding similar results, and the observed induction of TNKS-axin complexes was also confirmed in SW480 cells using the TNKSi XAV939 (S3B Fig).

### The proteasome is required for TNKSi induced assembly of axin-tankyrase-β-catenin complexes and formation of axin puncta

We next performed experiments to determine if blocking the proteasome with the inhibitor MG132 could, through stabilization of proteins, enhance the TNKS-axin complex formation seen with the TNKSi. Unexpectedly the converse was observed. We found that 6 h treatment with MG132 alone increased to some extent TNKS1 levels but had little effect on axin protein levels (Fig 3B) or the formation of axin-TNKS complexes (Fig 3A). In contrast, we discovered that co-treatment with MG132 completely blocked the ability of TNKSi (G007-LK) to induce binding of axin to TNKSs 1 and 2 (see Fig 3A and S3 Fig). This disruption of axin-TNKS complex formation correlated with reduced binding of β-catenin to axin, and suggests that an intact and active proteasome is required for assembly of axin-TNKS-β-catenin complexes.

**Fig 4:**
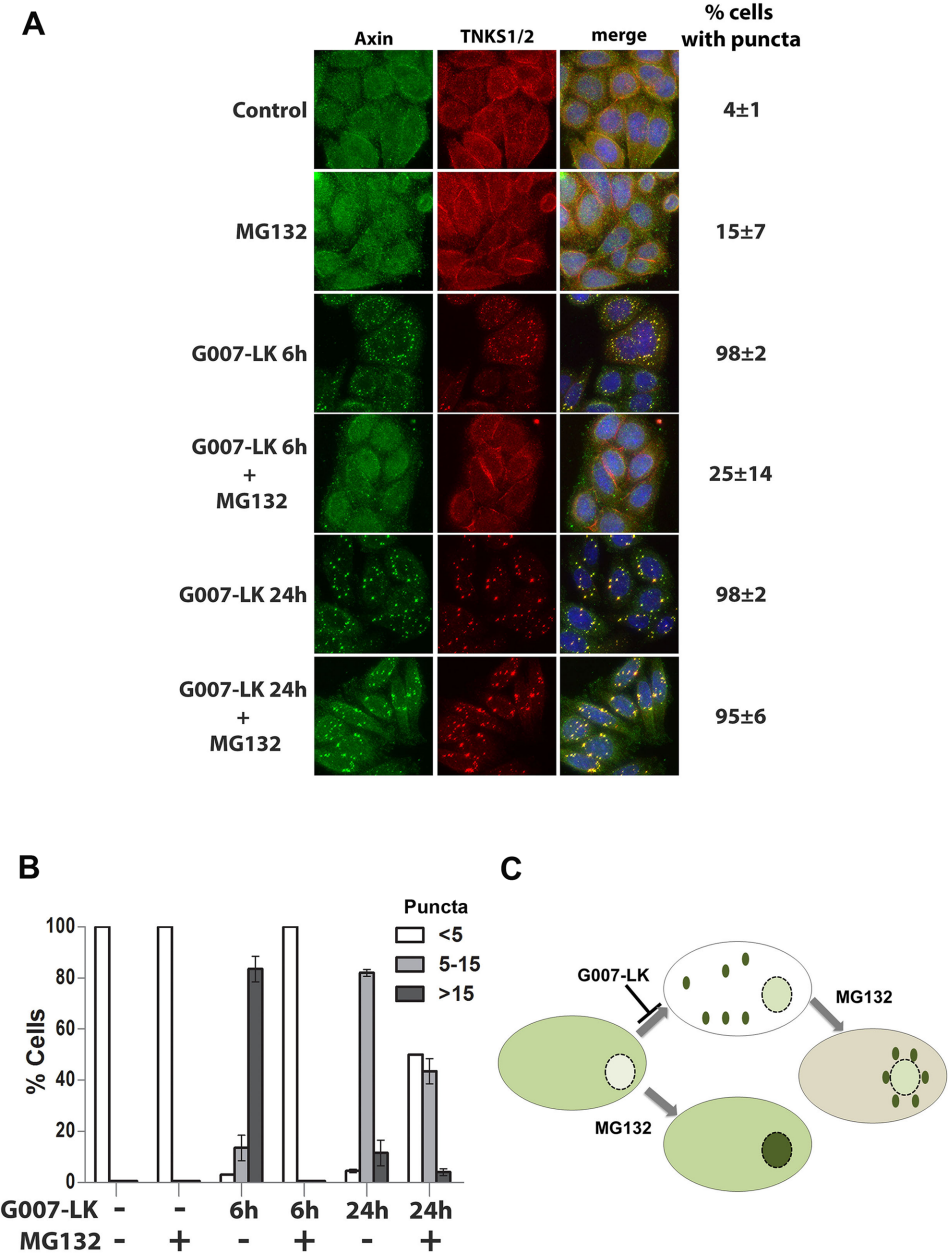
Induction of axin puncta by TNKSi is proteasome-dependent. **A.** SW480 cells were treated with single or combined doses of tankyrase (5 μM G007LK) and proteasome (20 μM MG132) inhibitors. The MG132 was added for 6 h, either simultaneously with G007-LK for a 6 h treatment, or during the last 6 h of a 24 h G007-LK treatment. As shown in the immunofluorescence images, the addition of MG132 caused a reduction in the % of cells with visible TNKSi-induced axin puncta after 6 h treatment. The 6 h MG132 treatment also caused a modest increase in nuclear staining of axin. The later addition of MG132 (at the end of a 24 h G007-LK treatment) caused the induced axin puncta to relocate to the perinuclear region. **B.** The number of axin puncta per cell were scored by microscopy and categorized (<5, 5–15 or >15 puncta per cell). As shown, TNKSi induced a high number of puncta per cell (more than 80% of cells scored >15 puncta per cell) and this was blocked at 6 h or reduced after 24 h by MG132 treatment. **C.** A diagram summarizing the effect of G007-LK +/- MG132 on axin pattern and cellular distribution (shown in green).

The above findings suggest that the proteasome might also be required for axin puncta formation. To address this hypothesis we treated SW480 cells with TNKSi for 6 h or 24 h either in the presence or absence of proteasome inhibitor, and examined cells for axin puncta by immunofluorescence microscopy. As shown in Fig 4A, treatment with MG132 for 6 h almost completely blocked the TNKSi induced formation of axin and TNKS-positive puncta (98% of cells presented visible axin puncta after TNKSi treatment, which decreased to 25% in presence of MG132). When the actual number of puncta per cell were analysed (Fig 4B), we found that MG132 had a large inhibitory effect after 6 h treatment, but still caused a measurable reduction in the number of TNKSi-induced puncta per cell after 24 h treatment (Fig 4B).

Interestingly, the treatment of MG132 for 6 h (Fig 4A) or in combination with TNKSi for up to 18 h (S4 Fig) appeared to shift the axin distribution to the nucleus, possibly by maintaining axin in a more soluble form. In contrast when MG132 was added after the axin puncta had formed (eg. MG132 added for the last 6 h of a 24 h TNKSi treatment) we found that the puncta relocated to the perinuclear region (Fig 4A). We were able to corroborate these results using different TNKSi and also a different proteasome inhibitor (S5 Fig), indicating that the effect is specific. These observations are summarized in a diagram (Fig 4C) showing axin redistribution under different treatments. The mechanism by which MG132 elicits a redistribution of the axin puncta is not yet clear, partly because so little is known of how axin puncta are positioned or relocated at specific sites within the cell. We can speculate that axin puncta positioning throughout the cytoplasm is, like many other large structures or organelles, at least partly influenced by microtubule-dependent motors. This idea is suggested by a previous report of insulin regulation of TNKS-axin-KIF3A complexes that were found to regulate GLUT4 transport [21]. Moreover it is intriguing that MG132 was reported to cause a localized block of microtubule-dependent transport of lysosomes and other organelles within the perinuclear region, in particular in a zone around the centrosome [22]. Therefore, perhaps MG132 treatment interferes with microtubule-dependent kinesin-mediated outward movement of axin puncta, causing them to drift toward the nucleus and accrue in the perinuclear zone. The actual details and mechanism await further investigation.

### Tankyrase inhibitors promote assembly of tankyrase and axin into large insoluble complexes

**Fig 5:**
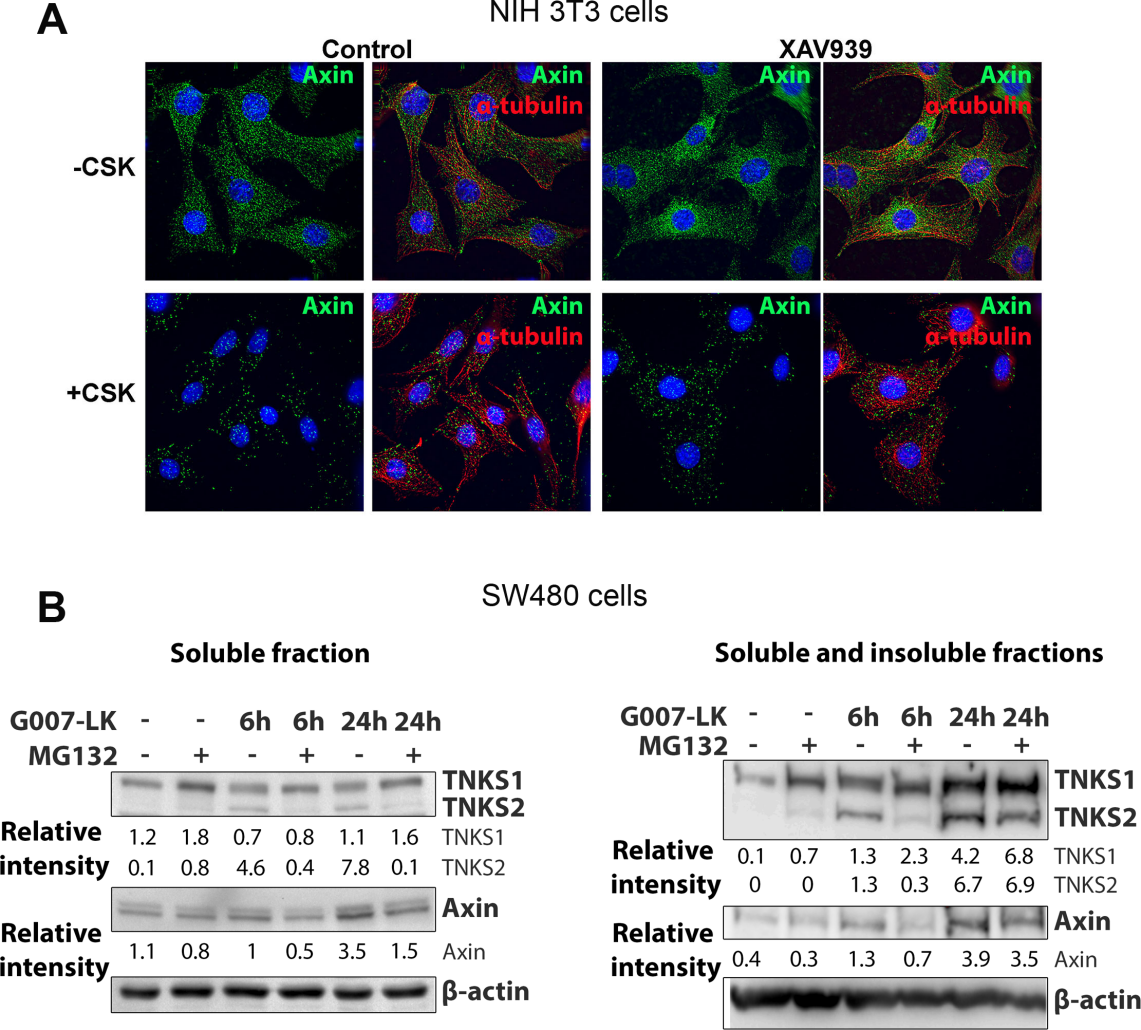
Tankyrase inhibitors promote inclusion of axin and TNKS into insoluble complexes. **A.** NIH 3T3 cells were left untreated (- CSK) or washed for 5 min with 0.2% Triton X-100 containing MT-buffer (+CSK) to permeabilize the plasma membrane and remove soluble proteins, before immunolabeling with fluorescent antibodies for axin and tubulin. Axin puncta in untreated cells and after induction by TNKSi were still visible after the detergent extraction, despite a decrease in background staining. This implies that the axin puncta are part of an insoluble pool resistant to extraction. **B.** To determine if axin and TNKS accrued in an insoluble fraction *in vitro* after TNKSi treatment in SW480 cells, cells were extracted either with RIPA buffer (left panel) or enriched for the insoluble fraction by extracting cells on the plate with SDS containing sample buffer (right panel). The addition of TNKSi (G007-LK) increased the accumulation of the TNKS-1 and -2 in the insoluble fractions and this was at least partly reduced for TNKS2 and axin following MG132 treatment, indicating that TNKSi might reduce solubility and mobility of axin and TNKS. Quantification of band intensity was measured and values were corrected to the endogenous control β-actin (a second experiment is shown in S6 Fig).

Recently it was proposed that TNKSi induce puncta at which TNKS remain tightly bound [9], compounding previous observations which showed that TNKS form insoluble complexes when oligomerized [7, 20]. We therefore investigated whether TNKSi-induced axin puncta were stable to detergent extraction and thus less prone to solubilization. We treated NIH 3T3 cells with TNKSi and extracted soluble proteins with detergent (+CSK), and under these conditions we also found that axin puncta remain quite stable and resistant to detergent extraction (Fig 5A). Next we harvested SW480 cells and prepared lysates using different extraction protocols. The standard extraction of total soluble protein revealed little effect of TNKSi on tankyrase expression but did confirm a modest increase in axin levels (Fig 5B) as seen earlier in Fig 1A. In contrast, a more robust extraction designed to collect insoluble protein complexes detected a clear increase in both TNKS, in particular TNKS-2 (S6 Fig), and axin pools (Fig 5B, S6 Fig). This is consistent with a recruitment of these factors into large oligomeric structures that form puncta. Treatment with proteasome inhibitor caused a slight reduction in TNKSi induced levels of TNKS-2 and axin in these insoluble complexes. This could possibly reflect a role for proteasome inhibition in reducing tankyrase-axin oligomer assembly and thereby provide one explanation for the positive effect of MG132 on axin movement to the nucleus (as described in Fig 4).

### TNKSi and an active proteasome maintain TNKS in a non-PARylated form, and this is reversed by MG132

**Fig 6:**
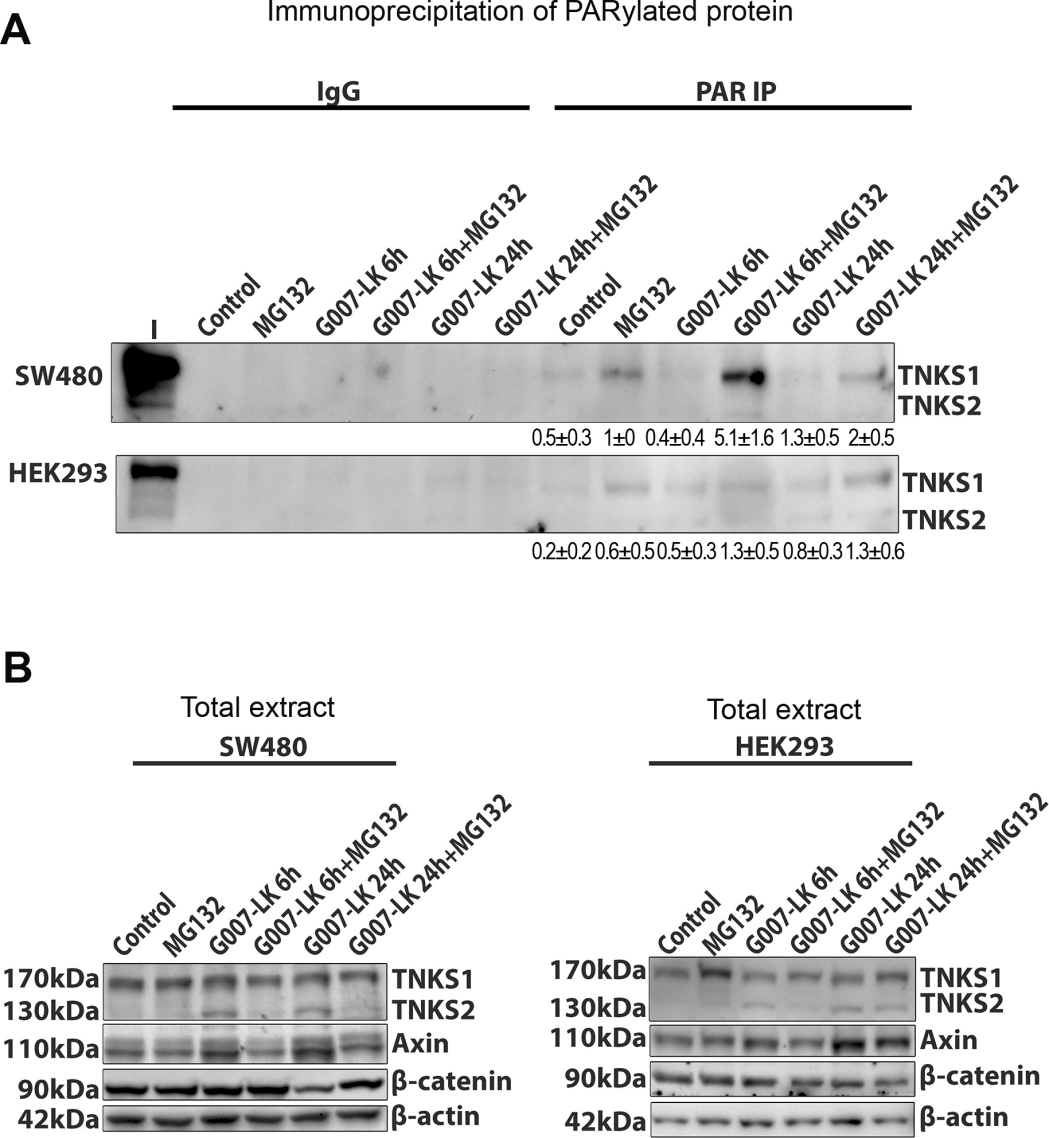
Proteasome inhibitor induces PARylation of TNKSs and TNKSi reduces basal PARylation levels. **A.** SW480 and HEK293 cells were exposed to combinations of TNKSi (G007-LK) and MG132 treatments similar to that shown in legend to Fig 3. The cells were then lysed and extracts analysed by IP assay using a specific antibody against PAR. PARylated forms of TNKS1/2 were then detected by western blot. The IgG control was negative. Pull-down of PARylated proteins revealed an increase in PARylation of TNKS1 by MG132 treatment, and a decrease of PARylation induced by TNKSi. When combined, the MG132 dominated and caused some induction of PARylated TNKS1 in both SW480 and HEK293 cells. Levels of PARylated TNKS2 (and also axin, not shown) were too low for detection. Quantification of band intensities (mean ± SD) was from two separate experiments. **B.** Total extract western blot shows similar TNKS levels after different treatments.

The TNKSi were reported to block the ability of TNKSs 1 and 2 to add poly(ADP)-ribose groups both to themselves and to axin [5, 7, 8, 13]. Moreover, both TNKSi and MG132 were reported to stabilize TNKS1 in MDCK epithelial cells, and in contrast to TNKSi, the MG132 elicited a particularly strong stabilization of the PARylated form of TNKS1 (see Fig 6B in [13]). We therefore tested the effects of TNKSi and/or MG132 on the PARylation of the TNKS using immunoprecipitation of PAR by specific antibody, followed by western blot to detect TNKS1 and 2. In SW480 cells, a faint band representing PARylated TNKS1 could be detected in untreated control cells and this increased after exposure to 6 h of proteasome inhibitor (Fig 6A). In contrast, treatment with TNKSi caused a slight reduction in PARylated TNKS1, as expected. The levels of PARylated TNKS2 and axin were too low for comparison in this assay. At 6 h and 24 h of G007-LK treatment, the co-treatment with MG132 appeared to dominate over the TNKSi, promoting an accumulation of PARylated TNKS1. This combination of drugs did not alter significantly TNKS or axin protein levels, indicating the specificity of this result (Fig 6B). Our findings confirm the previous observation of Yeh and colleagues [13] and indicate that proteasome inhibition stabilizes the PARylated form of TNKS1 effectively in SW480 cells. The ability of MG132 treatment to dominate over TNKSi might possibly be explained by MG132-mediated stabilization of another protein(s), such as a TNKS-binding partner, that can impede the de-PARylation caused by TNKSi.

### A model for the role of tankyrases in axin puncta formation

**Fig 7:**
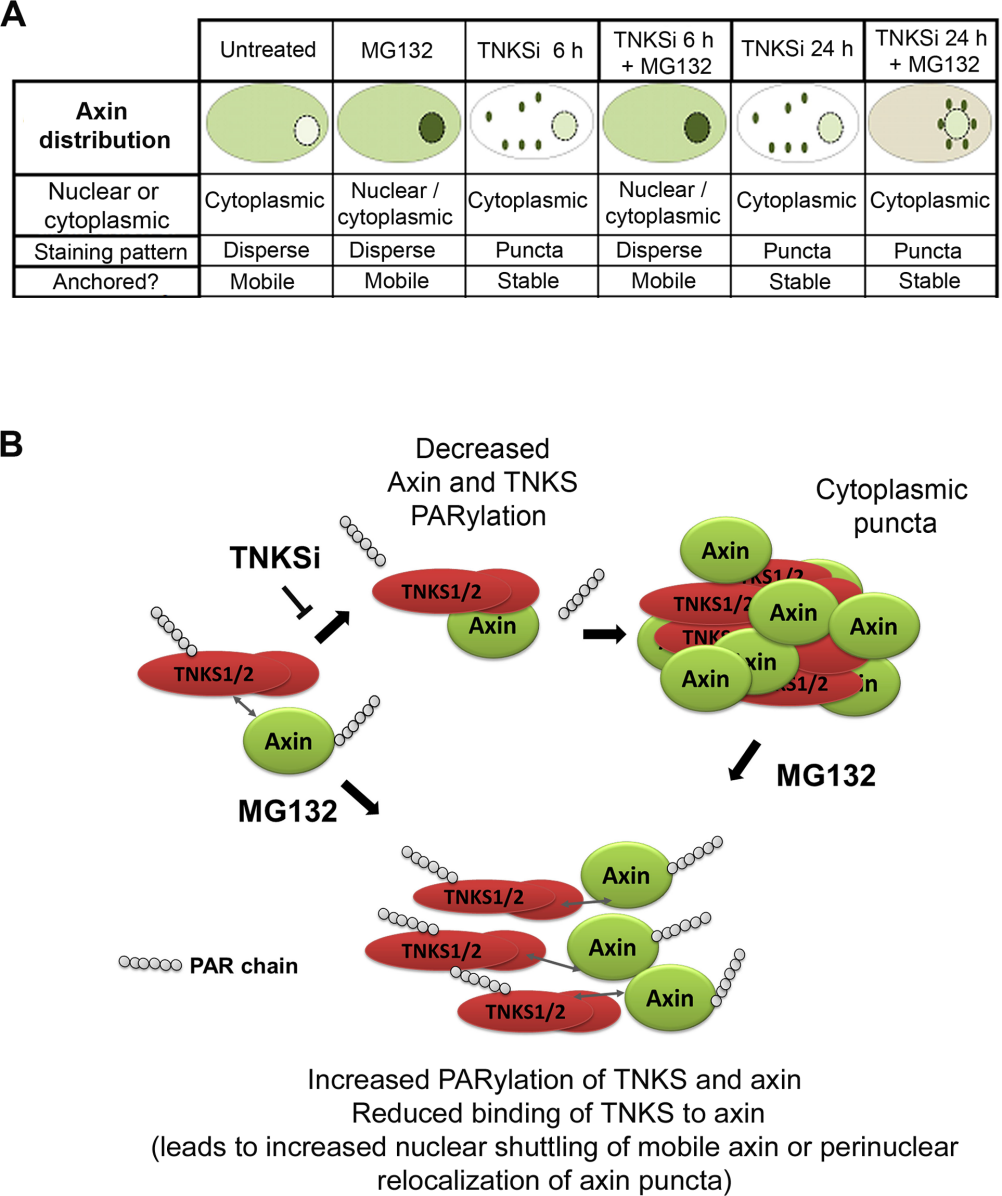
Model summarizing the action of TNKSi on formation of TNKS-axin complex formation and puncta assembly. **A.** Table with diagrams. **B.** Hypothetical model summarizing the effects of TNKSi on TNKS/axin binding, oligomerization and puncta formation by blocking PARylation of TNKS1. This model is strengthened by a consistent negative impact of MG132 on each of these different steps including the ability of TNKSi to dePARylate TNKS, induce axin-TNKS binding complexes and to induce cytoplasmic axin-TNKS puncta. The mechanism by which MG132, through proteasome inactivation, is able to block puncta formation occurs at least in part through stabilizing the PARylation of TNKS (and possibly axin), but is yet to be fully defined. We propose the following: treatment with MG132 alone stabilizes PARylated TNKS, which causes it to dissociate from axin leading to increased nuclear axin. Treatment with TNKSi alone can stabililize unPARylated TNKS which then binds axin and retains it in the cytoplasm in large complexes and puncta. When MG132 is added after TNKSi treatment, it will induce some increase in the pool of PARylated TNKS, and causes a perinuclear shift in the TNKS/axin puncta through a mechanism yet to be defined.

When considered together, our findings are consistent with a model in which TNKSi induce axin puncta/β-catenin degradation complexes at least partly through the de-PARylation of TNKS, which in turn leads to the promotion of TNKS oligomerization and the formation of axin-TNKS-β-catenin complexes. This is likely to contribute to the assembly of large axin/TNKS macromolecular complexes that generate puncta. This model is supported by previous literature showing that the PAR modification can inhibit TNKS oligomer formation and maintain the TNKSs in a more soluble state [7, 8]. It is also consistent with recent findings by Thorvaldsen et al. [9] who showed that overexpressed TNKS-GFP locates at TNKSi induced puncta that comprise TNKS-axin filamental complexes detected by electron microscopy. In addition, our results suggest that several of the key TNKSi-dependent steps in axin puncta formation require an active proteasome. A role for the proteasome is inferred indirectly by the ability of proteasome inhibitors to reduce axin puncta formation by (i) stabilizing the PARylated form of TNKS (Fig 6), (ii) reducing insoluble forms of TNKS and axin (Fig 5) and (iii) decreasing the TNKSi dependent formation of axin/TNKS/β-catenin complexes (Fig 3). These findings are summarized in Fig 7.

The mechanism of axin puncta formation and their role in coordinating the β-catenin degradasome complex [23] is an important topic in cancer research, largely due to the key role of β-catenin in cell transformation. Improved understanding of how TNKSi block aberrant wnt signaling in cancer cells is critical and may inform their potential usefulness when used in combination with other drugs. As confirmed in this study the TNKSi induce axin puncta in APC-mutant CRC cells, but it remains unclear whether β-catenin, following its N-terminal phosphorylation, is then ubiquitinated and degraded at puncta as suggested by Li et al. [24], or released for turnover elsewhere in the cytoplasm or plasma membrane as suggested by biochemical fractionation [25] and cell imaging [26], and to what extent or when this might occur. While the focus of this study was refined to analysis of TNKS and axin, there is evidence that APC is important for priming axin oligomerization [27] and that an interaction between the APC arm domain and axin may contribute to release of phospho-β-catenin under specific conditions [28]. TNKS act by adding large charged ADP-ribose modifications to proteins and such PARylation is known to cause dissociation and increased solubility of some protein complexes [10], including those containing TNKS [19, 20]. It will be important in future studies to determine how TNKS and its PARylating activity, combined with the subsequent effects on ubiquitination of substrates, contribute to the regulated interaction or disengagement of the various wnt component proteins within the puncta complex.

An interesting aspect to this study was the impact of proteasome inhibition, which effectively blocked the action of TNKSi on axin puncta formation at different stages. This is a novel result. Other studies have used MG132 for shorter time periods with overexpressed wnt components and observed some increase in N-terminal phosphorylated β-catenin at the puncta [9, 29], but reported no change in puncta formation. Our data suggest that the proteasome is important for cytoplasmic recruitment of axin to form axin-TNKS positive puncta, which reduces the normal shuttling of axin into the nucleus [30]. Active proteasome complexes locate throughout the cell but upon proteasome inhibition can congregate in the vicinity of the centrosome in structures termed aggresomes [31]. TNKS1 has also been detected at perinuclear sites including the golgi [32], centrosome and nuclear envelope [33]. We therefore speculate that under conditions of proteasome stress the axin/TNKS puncta may relocate close to the nuclear envelope where they merge into larger punctate structures (Fig 4) for possible degradation at aggresomes or related structures. We note that several proteasome inhibitors including MG132 have been used as therapeutic agents, alone or in combination with other agents, in colorectal cancer [34], and therefore it may be of relevance that our data suggest proteasome inhibitors counteract rather than potentiate the action of TNKSi drugs in CRC.

## Supporting Information

S1 Fig Effect of different TNKSi on axin puncta. SW480 cells were treated for 24 h with the TNKSi 2.5 μM XAV939 and 5 μM IWR-1. Immunofluorescence microscopy of endogenous axin in green shows the formation of cytoplasmic axin puncta after TNKS inhibition.
(JPG)

S2 Fig Effect of TNKSi-induced puncta in NIH 3T3 cells. NIH 3T3 control cells (left panel) or pβ-catenin-GFP (green) transfected cells (right panel) were treated with TNKSi for 24h (5 μM G007-LK) and then stained with antibodies against different β-catenin degradation complex components and analysed by microscopy. The control cells had very efficient turnover of β-catenin sometimes making detection of β-catenin degradation components difficult at axin puncta. Only after expressing ectopic β-catenin-GFP, was β-catenin, APC and GSK3β then frequently detected at axin puncta.
(JPG)

S3 Fig Confirmation that TNKSi induce binding of axin to TNKS. **A.** HEK293T cells were untreated or treated for 6 h and 24 h with 5 μM G007-LK (+/- 6 h with 20 μM MG132) and cell extracts were then harvested and subjected to immunoprecipitation (IP) and analysed as in Fig 3, and similar results were obtained. Right-hand panel shows a western blot of total protein extract and demonstrates that total levels of TNKS were not modified by drug treatments. **B.** SW480 cells were untreated or treated for 6 h and 24 h with 2.5 μM XAV939 (+/- 6 h with 20 μM MG132) and cell extracts were then harvested and subjected to immunoprecipitation (IP). These results are similar to those observed using the other TNKi in Fig 3. The right-hand panel shows a western blot of total protein extract demonstrating that total levels of TNKSs were not modified by drug treatments.
(JPG)

S4 Fig Prolonged MG132 treatment causes nuclear accumulation of axin. SW480 cells were treated simultaneously with 20 μM of MG132 and 5 μM of G007-LK for up to 18h. Cells were fixed and fluorescently stained for axin (green). Under these conditions, the co-treatment of MG132 completely blocked formation of TNKSi-induced axin puncta and instead promoted the translocation of axin to the nucleus.
(JPG)

S5 Fig Late addition of proteasome inhibitors redirects axin puncta to the perinuclear region. SW480 cells were treated with single or combined doses of tankyrase inhibitors (2.5 μM XAV939 and 5 μM IWR-1) and proteasome inhibitors (20 μM MG132 or 10 μM Bortezomib). The proteasome inhibitors were added for 6 h (MG132) or 4h (Bortezomib) toward the end of the 24 h TNKSi treatment. The data confirmed the MG132 results described in Fig 4. The later addition of proteasome inhibitors (at the end of a 24 h TNKSi treatment) caused the induced axin puncta to relocate to the perinuclear region, and quantifications are shown below images. Nucleus is stained blue with Hoechst chromatin dye.
(JPG)

S6 Fig TNKSi increase TNKS2 and axin levels in insoluble cell fraction. To confirm the data shown in Fig 5B, an alternate SW480 cell fractionation method was employed (see Methods) to separate soluble and insoluble fractions. The results showing TNKSi induction of TNKS2 and axin (less so of TNKS1) were very consistent with the data shown for in situ isolation of insoluble material in Fig 5. This experiment was repeated twice with similar results, and the band intensity of the TNKSs and axin are shown (normalised to actin control).
(JPG)

## Abbreviations

APC: adenomatous polyposis coli
CK1: casein kinase 1
CRC: colorectal cancer
GSK-3β: glycogen synthase kinase-3 beta
LEF-1: Lymphoid enhancer-binding factor-1
PAR: poly(ADP)ribose
PARP: poly(ADP)ribose polymerase
PARylation: poly(ADP)ribose addition
TCF: T-cell factor
TNKS: tankyrase
TNKSi: tankyrase inhibitors
