# Supplementary figures and images for: Tankyrase Inhibitors Stimulate the Ability of Tankyrases to Bind Axin and Drive Assembly of β-Catenin Degradation-Competent Axin Puncta
Source: PLoS One. 2016 Mar 1;11(3):e0150484. doi: 10.1371/journal.pone.0150484 (PMC4773256; doi:10.1371/journal.pone.0150484)

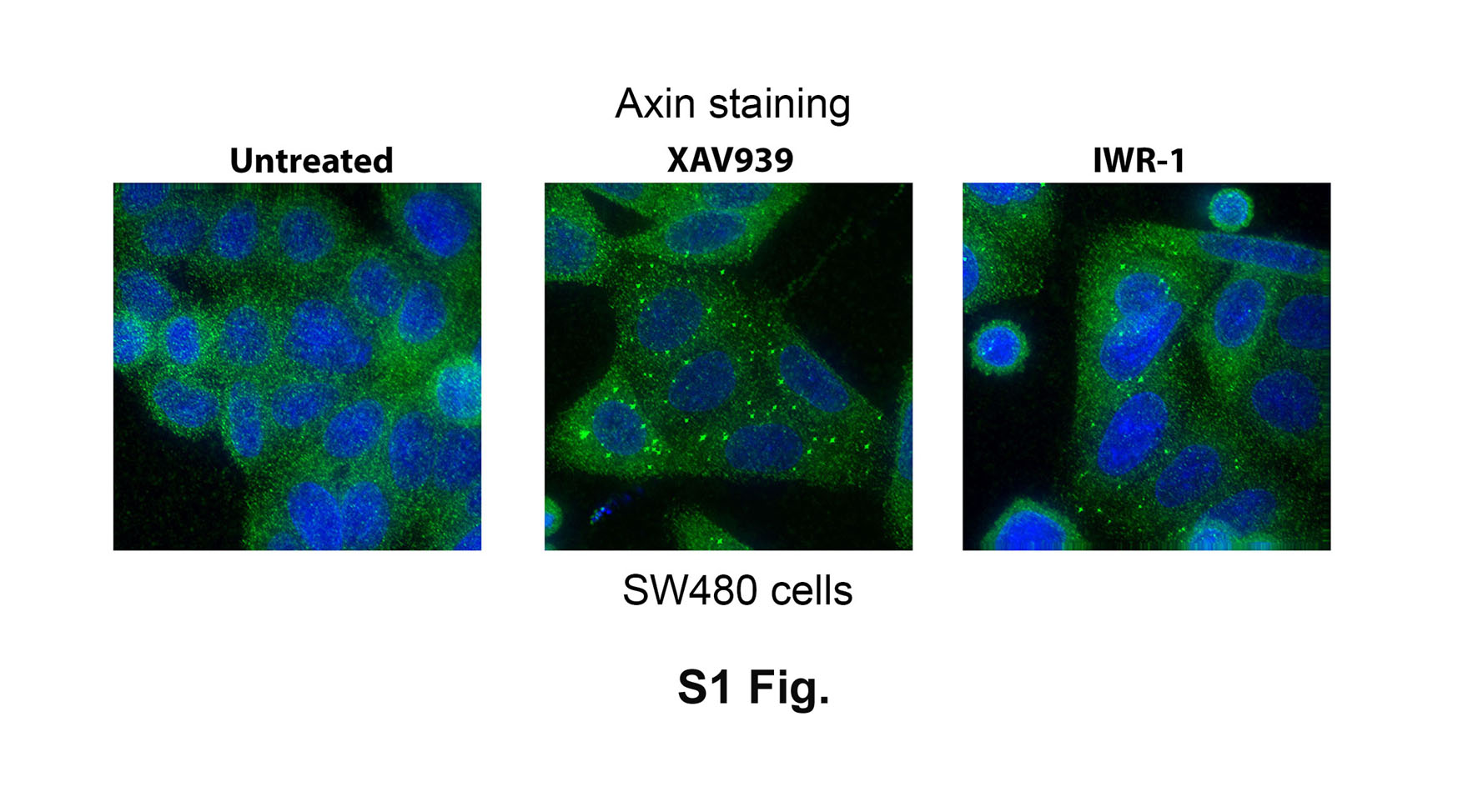

Supplement: S1 Fig — SW480 cells were treated for 24 h with the TNKSi 2.5 μM XAV939 and 5 μM IWR-1. Immunofluorescence microscopy of endogenous axin in green shows the formation of cytoplasmic axin puncta after TNKS inhibition. (JPG) [file pone.0150484.s001.jpg]

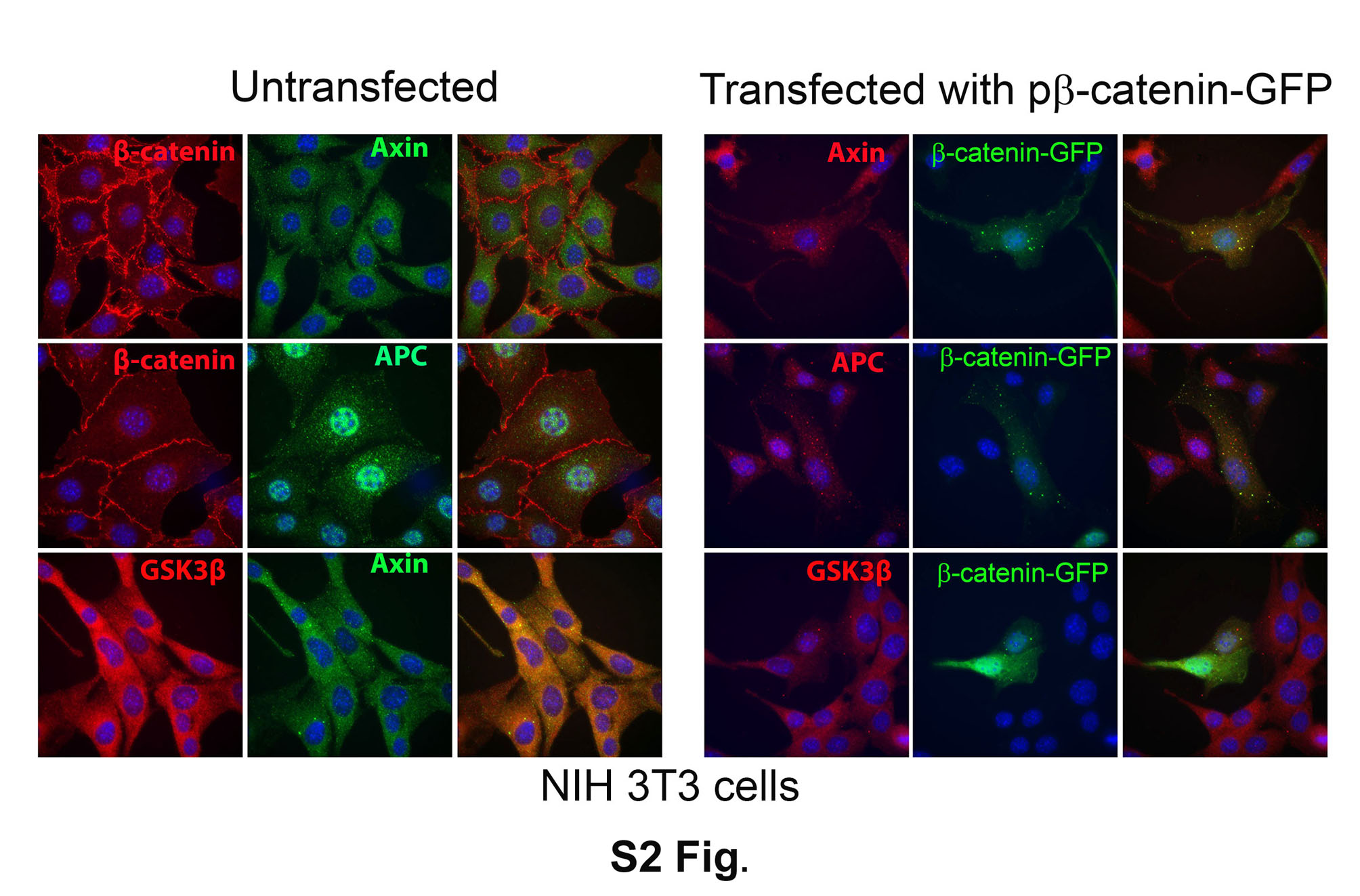

Supplement: S2 Fig — NIH 3T3 control cells (left panel) or pβ-catenin-GFP (green) transfected cells (right panel) were treated with TNKSi for 24h (5 μM G007-LK) and then stained with antibodies against different β-catenin degradation complex components and analysed by microscopy. The control cells had very efficient turnover of β-catenin sometimes making detection of β-catenin degradation components difficult at axin puncta. Only after expressing ectopic β-catenin-GFP, was β-catenin, APC and GSK3β then frequently detected at axin puncta. (JPG) [file pone.0150484.s002.jpg]

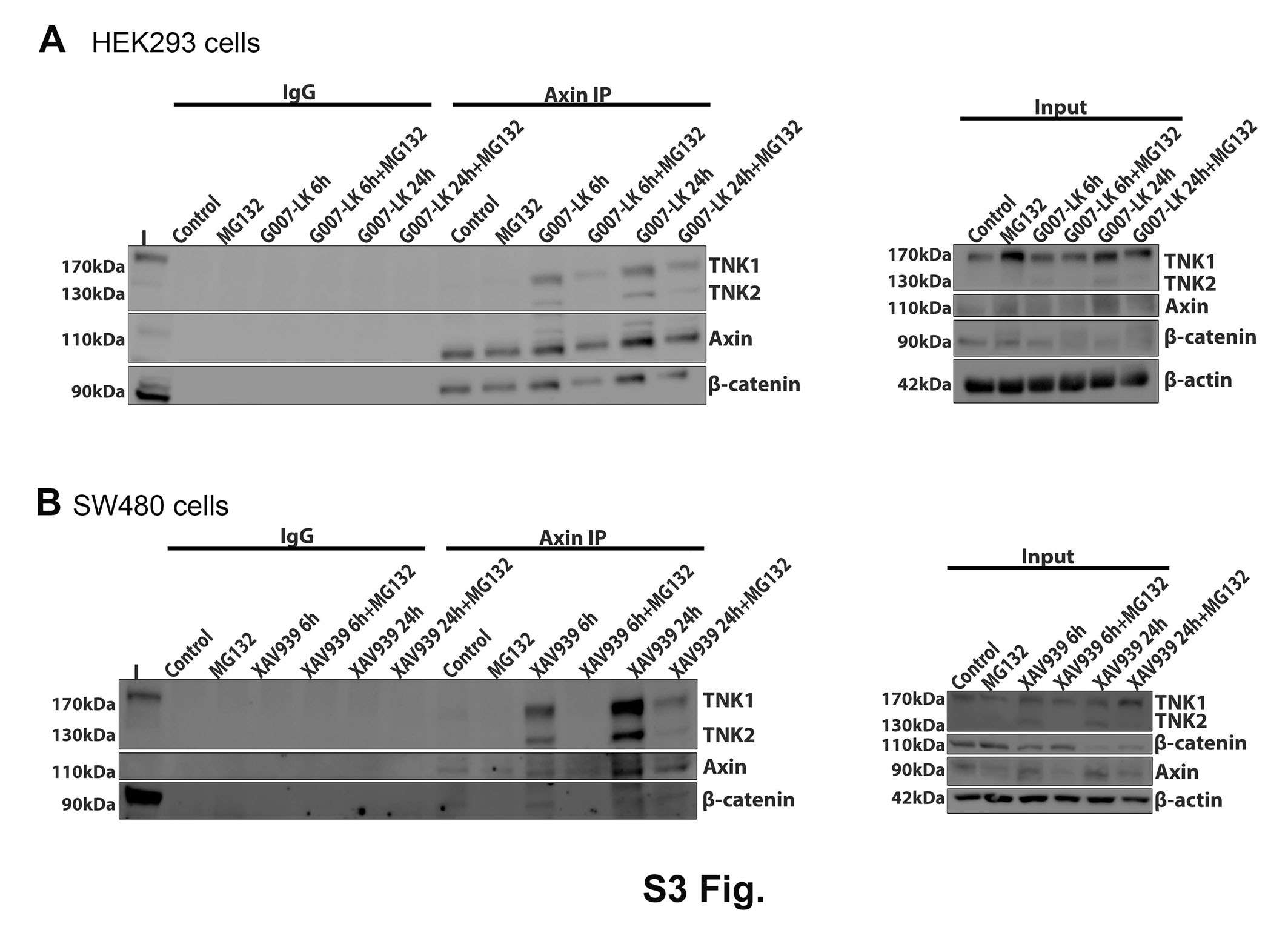

Supplement: S3 Fig — A. HEK293T cells were untreated or treated for 6 h and 24 h with 5 μM G007-LK (+/- 6 h with 20 μM MG132) and cell extracts were then harvested and subjected to immunoprecipitation (IP) and analysed as in Fig 3, and similar results were obtained. Right-hand panel shows a western blot of total protein extract and demonstrates that total levels of TNKS were not modified by drug treatments. B. SW480 cells were untreated or treated for 6 h and 24 h with 2.5 μM XAV939 (+/- 6 h with 20 μM MG132) and cell extracts were then harvested and subjected to immunoprecipitation (IP). These results are similar to those observed using the other TNKi in Fig 3. The right-hand panel shows a western blot of total protein extract demonstrating that total levels of TNKSs were not modified by drug treatments. (JPG) [file pone.0150484.s003.jpg]

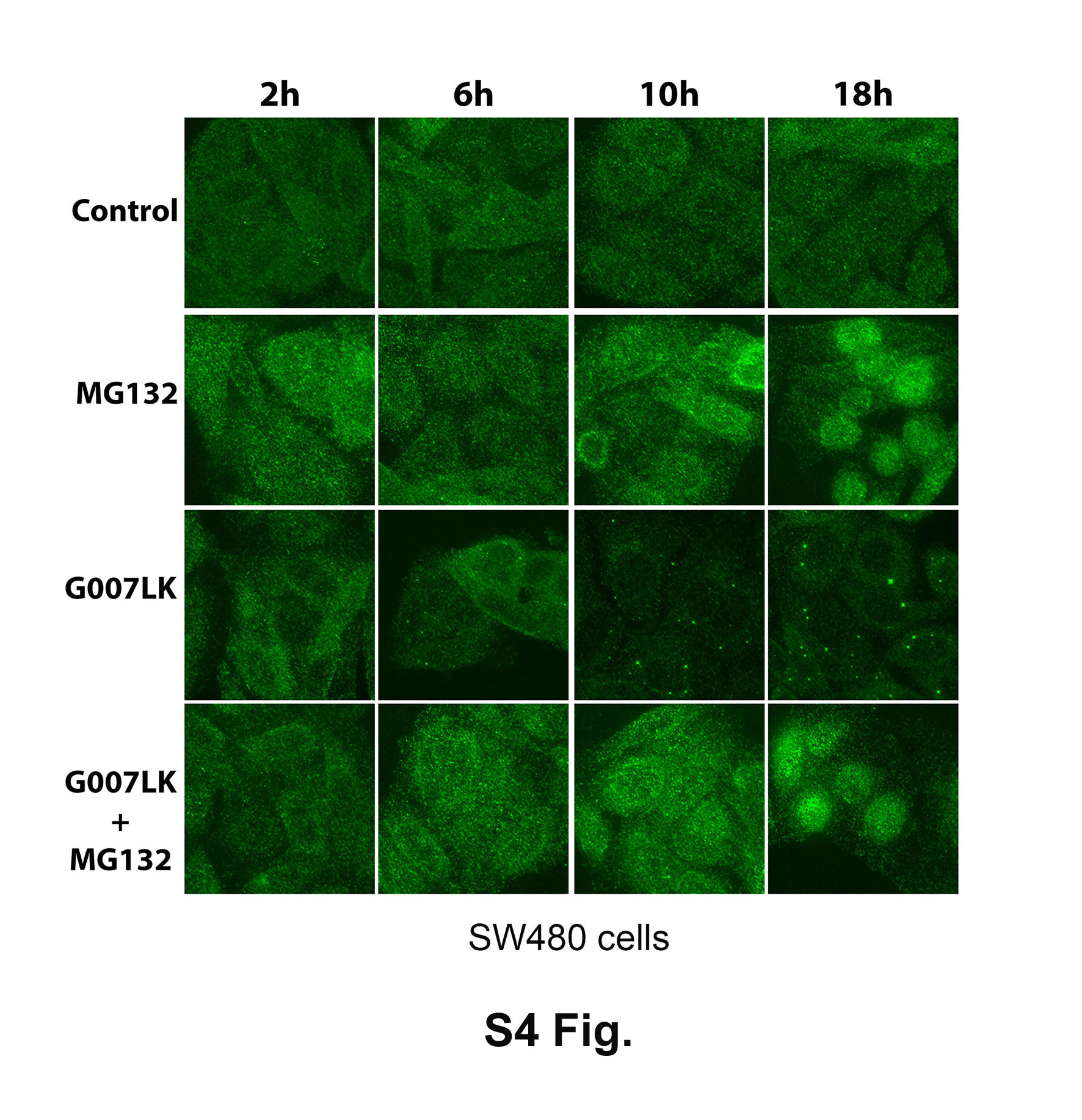

Supplement: S4 Fig — SW480 cells were treated simultaneously with 20 μM of MG132 and 5 μM of G007-LK for up to 18h. Cells were fixed and fluorescently stained for axin (green). Under these conditions, the co-treatment of MG132 completely blocked formation of TNKSi-induced axin puncta and instead promoted the translocation of axin to the nucleus. (JPG) [file pone.0150484.s004.jpg]

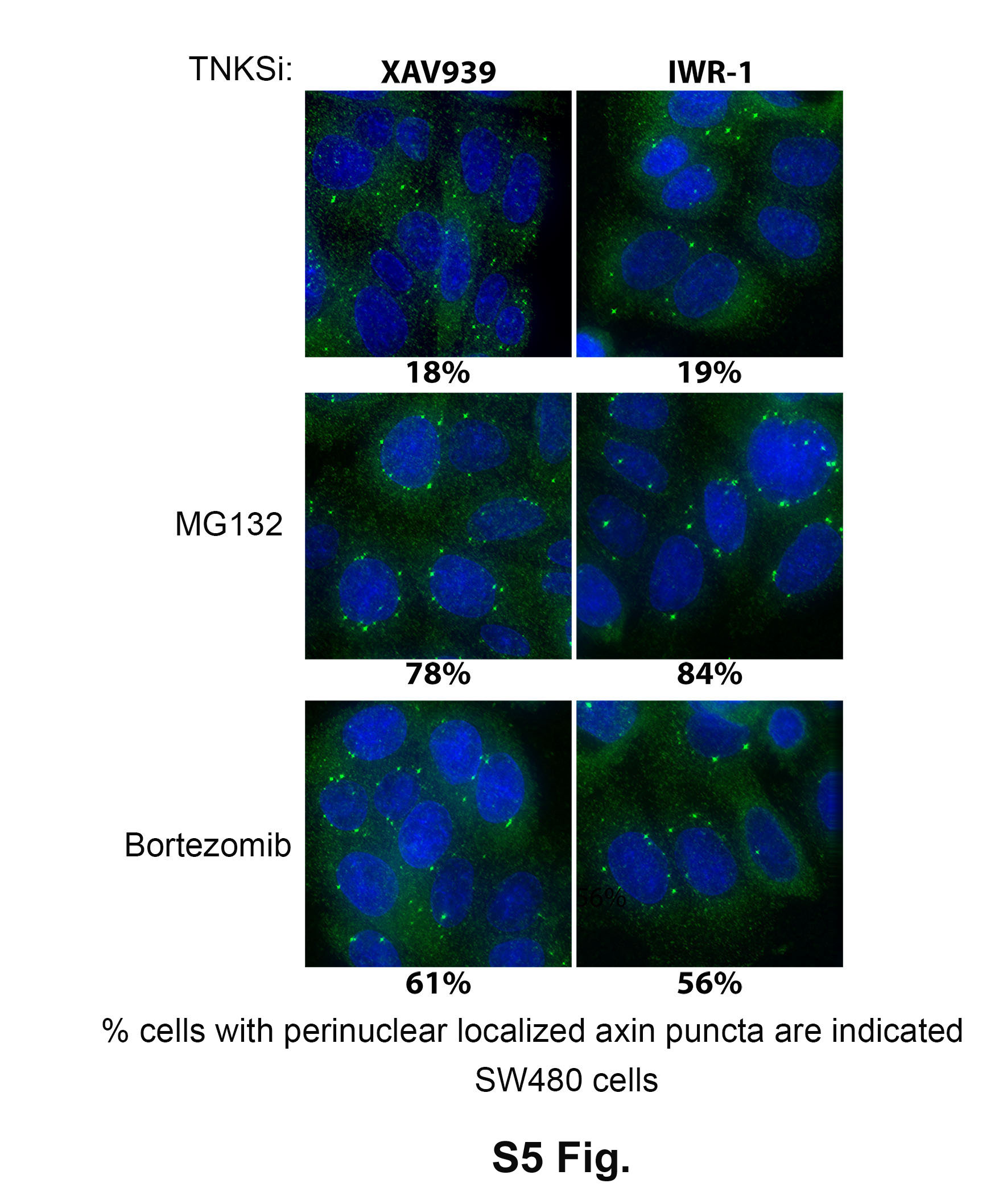

Supplement: S5 Fig — SW480 cells were treated with single or combined doses of tankyrase inhibitors (2.5 μM XAV939 and 5 μM IWR-1) and proteasome inhibitors (20 μM MG132 or 10 μM Bortezomib). The proteasome inhibitors were added for 6 h (MG132) or 4h (Bortezomib) toward the end of the 24 h TNKSi treatment. The data confirmed the MG132 results described in Fig 4. The later addition of proteasome inhibitors (at the end of a 24 h TNKSi treatment) caused the induced axin puncta to relocate to the perinuclear region, and quantifications are shown below images. Nucleus is stained blue with Hoechst chromatin dye. (JPG) [file pone.0150484.s005.jpg]

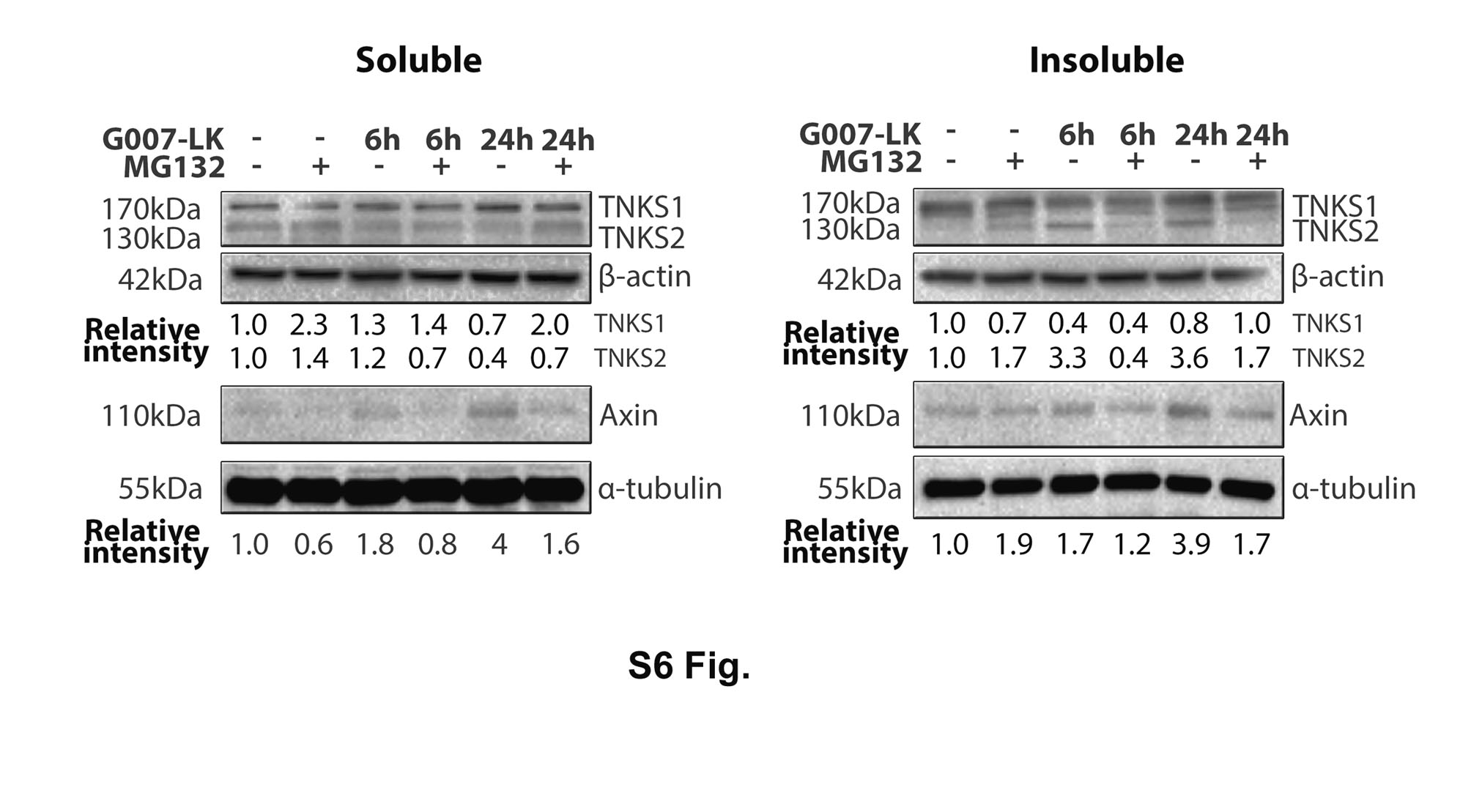

Supplement: S6 Fig — To confirm the data shown in Fig 5B, an alternate SW480 cell fractionation method was employed (see Methods) to separate soluble and insoluble fractions. The results showing TNKSi induction of TNKS2 and axin (less so of TNKS1) were very consistent with the data shown for in situ isolation of insoluble material in Fig 5. This experiment was repeated twice with similar results, and the band intensity of the TNKSs and axin are shown (normalised to actin control). (JPG) [file pone.0150484.s006.jpg]
